# 2-Hydroxybenzylamine (2-HOBA) to prevent early recurrence of atrial fibrillation after catheter ablation: protocol for a randomized controlled trial including detection of AF using a wearable device

**DOI:** 10.1186/s13063-021-05553-6

**Published:** 2021-08-28

**Authors:** Matthew J. O’Neill, Zachary T. Yoneda, Diane M. Crawford, Fei Ye, Mingfang Ao, Lisa M. Pitchford, John A. Rathmacher, Katherine T. Murray, Wendell S. Akers, Dan M. Roden, Gregory F. Michaud, M. Benjamin Shoemaker

**Affiliations:** 1 Vanderbilt University School of Medicine, Nashville, TN USA; 2 Department of Medicine, Division of Cardiovascular Medicine, Vanderbilt University Medical Center, Nashville, TN USA; 3 Department of Biomedical Statistics, Vanderbilt University Medical Center, Nashville, TN USA; 4 Department of Medicine, Division of Clinical Pharmacology, Vanderbilt University Medical Center, Nashville, TN USA; 5 MTI Biotech, Inc., Ames, IA USA; 6 Department of Kinesiology, Iowa State University, Ames, IA USA; 7 Department of Pharmaceutical Sciences, Lipscomb University College of Pharmacy, Nashville, TN USA; 8 Department of Biomedical Informatics, Vanderbilt University Medical Center, Nashville, TN USA

**Keywords:** Atrial fibrillation, Ablation, Pulmonary vein isolation, Isolevuglandin, 2-HOBA, Reactive oxygen species, Apple Watch, Chromosome 4q25, *PITX2*

## Abstract

**Background:**

Although catheter ablation is an effective therapy for atrial fibrillation (AF), the most common cardiac arrhythmia encountered in clinical practice, AF ablation generates inflammation and oxidative stress in the early postoperative period predisposing to recurrence of AF. Isolevuglandins (IsoLGs) are reactive lipid mediators of oxidative stress injury that rapidly react with endogenous biomolecules to compromise their function. 2-Hydroxybenzylamine (2-HOBA), a potent small molecule scavenger of IsoLGs, sequesters the reactive species as inert adducts. This mechanism, coupled with reported safety in humans, supports the investigation of 2-HOBA as a novel therapeutic to reduce AF caused by oxidative stress, such as that which occurs after catheter ablation. Accordingly, we seek to test the hypothesis that treatment with 2-HOBA will decrease early recurrence of AF and other atrial arrhythmias following AF ablation by decreasing IsoLG adducts with native biomolecules.

**Methods:**

The proposed trial will randomly assign 162 participants undergoing cryo- or radiofrequency catheter ablation for AF to 2-HOBA (*N* = 81) or placebo (*N* = 81). Individuals will begin the study drug 3 days prior to ablation and continue for 28 days. Participants will be given a wearable smartwatch capable of detecting and recording atrial arrhythmias. They will be instructed to record ECGs daily with additional ECGs if they experience symptoms of AF or when alerted by the smartwatch AF detection alarm. The primary clinical endpoint will be an episode of AF, atrial tachycardia, or atrial flutter lasting 30 s or more within 28 days post-AF ablation. Secondary measures will be the change in IsoLG adduct levels from blood samples collected immediately pre-ablation and post-ablation and reduction in AF burden as calculated from the smartwatch.

**Discussion:**

The proposed trial will test the hypothesis that 2-HOBA reduces post-ablation atrial arrhythmias through sequestration of reactive IsoLG species. The results of this study may improve the understanding of the role of IsoLGs and oxidative stress in AF pathogenesis and provide evidence to advance 2-HOBA and related compounds as a new therapeutic strategy to treat AF.

**Trial registration:**

ClinicalTrials.gov NCT04433091. Registered on June 3, 2020.

## Administrative information

The order of the items has been modified to group similar items (see http://www.equator-network.org/reporting-guidelines/spirit-2013-statement-defining-standard-protocol-items-for-clinical-trials/).

**Table:** 

Title {1}	2-Hydroxybenzylamine (2-HOBA) to Prevent Early Recurrence of Atrial Fibrillation after Catheter-based Ablation: Protocol for the Randomized Controlled Trial Including Detection of AF Using a Wearable Device
Trial registration {2a and 2b}.	NCT04433091
Protocol version {3}	Version 1 – June 16th, 2020
Funding {4}	American Heart Association Strategically Focused Atrial Fibrillation Research Network Award to Vanderbilt University Medical Center (AHA 18SFRN34110369)
Author details {5a}	Vanderbilt University Medical Center – Department of Medicine Division of Cardiovascular Medicine, Nashville, TN, USA
Name and contact information for the trial sponsor {5b}	Gregory Michaud, MD (gregory.f.michaud@vumc.org)
Role of sponsor {5c}	This is an investigator-sponsored study funded by the American Heart Association with study drug (2-HOBA) and placebo donated by MTI Biotech, Inc. (Ames, IA, USA). Final decisions related to the study design, data collection, analysis, interpretation, and manuscript preparation were made by the investigator-team.

## Introduction

### Background and rationale {6a}

Atrial fibrillation (AF) is the most common arrhythmia in clinical practice and is associated with an increased risk of stroke, heart failure, and myocardial infarction. Current treatment options to maintain sinus rhythm include antiarrhythmic drugs (AADs) and catheter-based AF ablation. Unfortunately, AADs are associated with a narrow therapeutic window, cardiac and non-cardiac side effects, and only modest long-term efficacy. The alternative, catheter ablation, is an invasive procedure with inherent procedural risks, and while highly effective in some patients, it may be ineffective in others and/or require multiple procedures. Therefore, the development of new medical options for the treatment of AF represents a major unmet need [1].

Inflammation and resulting oxidative stress underlie the pathogenesis of many disorders, including AF. The oxidation of polyunsaturated fatty acids by reactive oxygen species (ROS) generates a variety of highly electrophilic molecules, including IsoLGs [2]. IsoLGs irreversibly sequester biological nucleophiles, especially primary amines such as lysine, thereby forming permanent adducts that compromise protein function. Small molecule scavenging of these reactive species to form inactive adducts has thereby emerged as an attractive therapeutic strategy. 2-Hydroxybenzylamine (2-HOBA) is a natural product present in buckwheat that reacts rapidly with IsoLGs [3]. This biological activity has demonstrated efficacy in preclinical studies across diverse models of oxidative stress, including AF [4] and Alzheimer’s disease [5]. Phase I single-dose study (NCT-3176940) [6] and multidose (NCT-03555682) [7] studies were conducted to assess the compound’s safety and develop a pharmacokinetic profile in 41 participants. The drug was well tolerated, with a similar side effect profile among participants taking 2-HOBA and those taking placebo. Given the novel mechanism and excellent safety profile, 2-HOBA has emerged as a promising drug candidate to treat AF by reducing oxidative stress injury.

Myocardial injury from AF ablation generates sterile inflammation and oxidative stress in the first several months immediately following the procedure, leading to early recurrence in up to 50% of patients [8]. Early recurrence of AF has been associated with an increased risk of long-term recurrence [9]. Studies investigating treatments to reduce early recurrence have demonstrated the efficacy of corticosteroids [10] and colchicine [11], but both suffer from long-term side effects that limit widespread use. We therefore propose a phase II trial to study the ability of 2-HOBA to reduce the early recurrence of AF after catheter ablation via a novel mechanism to reduce IsoLGs in the post-procedure period typically characterized by high levels of inflammation and oxidative stress.

We hypothesize that the administration of 2-HOBA in patients undergoing AF ablation will reduce the recurrence of AF within 28 days following ablation. We will enroll 162 participants to receive either placebo (*N* = 81) or 2-HOBA (*N* = 81) in a single-center, double-blind, randomized clinical trial. Our primary outcome will be at least 30 s of AF, atrial flutter, or atrial tachycardia that occurs within 28 days following AF ablation as recorded using a smartwatch.

### Objectives {7}

Objective #1 (the clinical aim) will test the ability of 2-HOBA to reduce atrial arrhythmias. The primary outcome for this will be the early recurrence of AF, atrial flutter, or atrial tachycardia (yes or no) in the 4 weeks following an AF ablation procedure. The secondary outcome will be the burden of atrial arrhythmias as measured using a smartwatch. The burden will be defined here as the proportion of time in an atrial arrhythmia over the total time monitored. Objective #2 (the biochemical aim) will test the ability of 2-HOBA to reduce IsoLG adducts. Objective #3 (the exploratory genomic aim) will test whether common genetic variation at chromosome 4q25 (*PITX2*), a gene involved with the oxidative stress response [12], is associated with AF recurrence or IsoLG adduct levels. These complementary clinical, biochemical, and genomic endpoints will be assessed through the trial framework described below.

### Trial design {8}

This will be a double-blind, randomized, parallel design clinical trial comparing 2-HOBA (*N* = 81) vs placebo (*N* = 81) for the reduction of clinical AF and biochemical IsoLG adduct levels in participants undergoing AF ablation at Vanderbilt University Medical Center. Eligible participants will be randomized 1:1 according to a stratified permuted block scheme with a block size of 8 via a webtool, with participants and trial personnel blinded to the treatment assignment. The primary outcome will be 30 s of AF, atrial flutter, or atrial tachycardia as detected by a wearable, single-lead ECG within the first 28 days following ablation. The trial will be powered to detect superiority in the intervention arm over the placebo arm for the clinical outcome.

## Methods: participants, interventions, and outcomes

### Study setting {9}

The trial will be completed entirely within the Vanderbilt Heart and Vascular Institute at Vanderbilt University Medical Center (VUMC, Nashville, TN, USA), the largest academic hospital system in Tennessee. All ablations will be performed by VUMC electrophysiologists in the EP lab, and participants will receive their pertinent post-ablation follow-up care at VUMC or one of the affiliated hospitals in Bedford, Davidson, Maury, Montgomery, Rutherford, Sumner, or Williamson County, TN.

### Eligibility criteria {10}

Participants must be aged 22 or older at the time of the trial, undergoing ablation for the first time with radiofrequency or cryoablation or repeat ablation for persistent AF when ablation of non-pulmonary vein substrate is planned (e.g., posterior wall ablation, mitral or roof line). Participants will be required to provide written, informed consent to be eligible to participate in this trial.

The exclusion criteria will include planned surgical or hybrid ablation, amiodarone use currently or within the past 3 months, use of oral steroids or colchicine prior to ablation, pro-inflammatory and rheumatologic disorders, NYHA class III/IV HF, active ischemia, hypertrophic cardiomyopathy, cardiac or thoracic surgery within the last 6 months, life expectancy < 1 year, creatinine clearance < 30 ml/min, prior or planned heart transplantation, pregnancy, aspirin allergy, or current use of MAO inhibitors.

### Who will take informed consent? {26a}

Eligible participants will be identified through the electronic medical record after being scheduled for an AF ablation. A member of the study team (study nurse or physician), not otherwise associated with clinical care for the participant, will request an introduction from the clinical staff. If the participant agrees, a member of the study team will approach the participant in person in the Arrhythmia Clinic or by phone prior to AF ablation. Information describing the study, why the research is being done, and what will be learned will be provided. The risks as described in this protocol will be delineated. The benefits will be described as general scientific knowledge, along with potential treatment advances. No direct immediate benefit to the participants is anticipated. Informed consent will be documented with the subject’s signature using an Institutional Review Board (IRB)-approved consent form for this protocol. An option will be available for phone consent with an electronic signature.

### Additional consent provisions for collection and use of participant data and biological specimens {26b}

All records will be retained on password-protected computers accessible only to members of the study team. Computers containing these records are only connected to the networks if they include appropriate firewalls and security measures. Deidentified records and DNA samples may be shared with other investigators who have IRB-approved protocols and who agree to comply with the protections provided at this institution. These research materials will be transferred only by secure methods. The identity of any individuals and their families will not be revealed in any publication without their written informed consent. An approved genetic rider from VUMC is included in the informed consent paperwork and can be found as a link here (VUMC Genetic Rider).

### Interventions

#### Explanation for the choice of comparators {6b}

2-HOBA is chosen for the study based on its high potency to scavenge IsoLGs in mouse models and excellent safety profile in human clinical trials, supporting the hypothesis that the administration can diminish rates of AF recurrence in humans in the post-ablation setting. 2-HOBA is a natural product that is approved for sale in the USA at lower doses as a dietary ingredient, supporting its status as a safe therapeutic. Its pharmacokinetic properties are acceptable for human use, with TID dosing of 750 mg achieving steady-state concentration anticipated to be effective. The half-life is 3.1 h with repeated dosing, and 2-HOBA is metabolized to salicylic acid. 2-HOBA has not been tested against placebo for the management of any disease or condition in humans to date. Anti-inflammatory strategies have been tested for the management of AF using steroids and colchicine [10, 11]. Both have been shown to reduce AF recurrence following ablation; however, the adverse side effect profiles of colchicine and steroids prohibit their use as long-term therapies. Given the safety profile of 2-HOBA in phase I studies, 2-HOBA is expected to have a better side effect profile for continuous long-term use. As the efficacy of the medication has not been established, we will use a placebo in this trial as a comparator for both the safety and efficacy of this novel strategy.

#### Intervention description {11a}

Participants will be randomized to receive 2-HOBA acetate (750 mg) or placebo orally every 8 h for 3 days before AF ablation, with continued dosing for 28 days after ablation. In its current formulation, 2-HOBA acetate comes as 250-mg capsules; the dosing for this trial will be 3 capsules of study drug (2-HOBA or placebo), three times per day taken by mouth. Participants will be instructed to take the first three capsules of the study drug at 6 AM.

#### Criteria for discontinuing or modifying allocated interventions {11b}

Participants may withdraw from the study at any time by informing the study staff verbally or in writing. No intervention modification will be planned. Based on human studies to date, the number of serious adverse events (SAEs) is expected to be minimal. Should an SAE occur that is felt to be related to the study drug, the drug will be discontinued. As the study will be double-blinded, neither participants nor study personnel will know if a participant is on placebo or 2-HOBA; requests to unblind or switch treatment assignments will not be granted. There is no current standard of care for the treatment of AF recurrence within the first 28 days following ablation; in the field of AF ablation, recurrence within the first 90 days following ablation is considered to be in the “blanking period” and does not represent a long-term failure of ablation. Clinical treatment for recurrence within the first 28 days following the ablation will be at the discretion of the clinical provider. As 2-HOBA is currently not approved for use in any disease or condition, crossover as a result of the clinical discretion of the provider will not be possible. A participant may be withdrawn from the study by the PI if any of the following occurs: (1) the participant, for any reason, does not undergo catheter ablation; (2) the participant does not comply with the study protocol, such as failure to attend research encounters or inability to wear the smartwatch; and (3) the participant experiences an SAE felt related to the study drug.

#### Strategies to improve adherence to interventions {11c}

Daily rhythm monitoring with the wearable device immediately after taking the morning medication dose will be required for the participant. The wearable device records all ECG strips taken, and at the end of the trial, the number of strips obtained will be recorded and documented. An IRB-approved worksheet with instructions for taking the medication as well as a table to allow the participant to record symptoms, number of pills taken, and number of ECG recordings taken per day will be given to all participants. Participants will be given the study coordinator’s phone number and email address to contact if they have any concerns or questions. To further document adherence, in addition to filling out the worksheet, the participant’s remaining pills will be counted to check if the appropriate number of pills was taken. Finally, blood will be drawn from participants on the day of the ablation (3 days after starting the intervention), the day after the ablation (4 days after starting the intervention), and 28 days after the ablation (31 days after starting the intervention). A portion of this sample will be sent for drug levels both to determine pharmacokinetic properties and to assess adherence. The timing of the last dose of the study drug and blood draws are recorded. The study staff responsible for participant interaction and determination of the primary clinical and biomarker endpoints will remain blinded to the results of the study drug level analyses.

#### Relevant concomitant care permitted or prohibited during the trial {11d}

All participants will undergo catheter ablation consisting of a pulmonary vein isolation (PVI) 3 days after the start of the study drug intervention. Further adjunctive lesions placed as part of the ablation will remain at the discretion of the clinical team. AF ablation will be an essential component of this study, providing the necessary oxidative stress to test the efficacy of 2-HOBA for reducing the clinical and biochemical endpoints. The pulmonary vein myocardial sleeves are a proven source of AF triggers that can be treated by electrically isolating them from the left atrium through the creation of circumferential ablation lines. Currently, the location of the pulmonary veins is identified by a variety of imaging modalities including fluoroscopy, electroanatomic mapping, and/or intracardiac ultrasound. Ablation lesions are applied circumferentially around the pulmonary vein ostia. This is an example of an anatomic, or substrate-guided, ablation approach, and it does not require the induction of an arrhythmia to perform. Induction of AF is not a procedural endpoint, so the potential of 2-HOBA to decrease AF inducibility does not affect the procedure. The main procedural endpoint is the demonstration of electrical isolation of the pulmonary veins.

Participants with AF undergoing ablation are often on AADs at the time of their ablation. Amiodarone use within 3 months including concurrent amiodarone use will be an exclusion criterion for this trial. Anticoagulation of participants undergoing ablation is common prior to the ablation and required for all participants after ablation. This trial will not change this clinical guidance. Potential participants on colchicine or corticosteroids prior to enrollment will be excluded from participation. Colchicine use following the ablation will be regulated; if colchicine is prescribed following the ablation due to a clinical indication (i.e., pericarditis), this trial will not prohibit its use. Colchicine use prophylactically following ablation will be discouraged. The use of immunosuppressive drugs following the procedure will be documented. Any change of AF-related therapy (discontinuation of anticoagulation, change of anti-arrhythmic medication, cardioversion, hospitalization, or repeat ablation) will be documented, but permitted in this trial.

#### Provisions for post-trial care {30}

Follow-up at 1 month and 3 months will take place at the Vanderbilt Arrhythmia Clinic for all participants as a standard component of post-ablation care. Follow-up typically occurs at 1 month and 3 months. No additional study visits will be planned as a consequence of this trial; trial visits will be coordinated with the 1-month clinical follow-up visit. In the case of injury, Vanderbilt will pay for the cost of immediate care to treat the injury but will not cover any additional care or provide financial compensation.

### Outcomes {12}

The primary outcome of this study will be AF, atrial flutter, or atrial tachycardia lasting 30 s within the 28 days following ablation as recorded by single-lead ECG recording on the wearable device. Digital smartwatches (Apple Watch, Apple Inc., Cupertino, CA) can be used to automatically detect cardiac arrhythmias such as AF [13] and to record an on-demand single-lead ECG. Participants will be instructed to record a rhythm strip every morning and for the following: (1) should the device notify the participant of a sustained, elevated heart rate (greater than 100 beats per minute), (2) should the device notify the participant of an irregular rhythm, or (3) should the participant have symptoms of AF. At the end of the 28-day period, the rhythm strips will be available for review. The study personnel will review all ECG tracings to adjudicate the primary outcome. Thirty seconds or more of documented AF/atrial flutter/atrial tachycardia is often used as the primary outcome for rhythm control strategies [14]. In the case of AF ablation, this outcome is ascertained after a 90-day “blanking” period due to the transient inflammation that occurs. This trial will use the same primary outcome with follow-up limited to the first 28 days following ablation to increase the anticipated number of events. This move will also decrease the necessary follow-up time for earlier validation of a novel mechanism prior to a larger trial.

This trial will have two pre-defined secondary outcomes. The first will be an exploratory clinical outcome of AF burden. AF burden is the fraction of time any given participant spends in AF, atrial flutter, or atrial tachycardia. Although this outcome may have more clinical relevance [15], it is not typically used in rhythm control or ablation trials due to a lack of continuous monitors. This trial will involve the use of a wearable technology for which burden has not previously been calculated. We propose a novel method of calculation of AF burden by the summation of the length of time a participant is notified that they have a high heart rate and the episodes in which a participant is notified that they have an irregular rhythm, divided by the total wear time for the 28-day period.

The biochemical secondary outcome is the quantitation of IsoLG adduct levels in dendritic cells. These measurements will be made using flow cytometry of blood samples pre-ablation and on post-op day #1. Levels of IsoLG adducts will be measured before and after ablation to assess for variable changes in the levels between participants receiving 2-HOBA or placebo. The increase will be reported as a mean for the intervention vs the placebo group and compare pre- and post-ablation adduct levels.

#### Participant timeline {13}

**Fig. 1:**
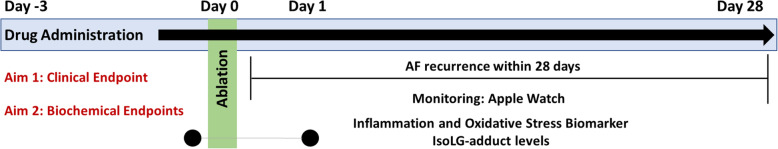
Participant timeline

After obtaining informed consent, study activities will be conducted according to the timeline in Fig. 1.

#### Sample size {14}

The sample size for this study is based on the binary primary endpoint (30 s of AF as detected by single-lead ECG) of early AF recurrence by the end of follow-up. A total sample size of 162 with 81 in each arm will provide 81% power to detect a reduction in the rate of early recurrence of AF if the event rate is 29% in the treatment arm and the rate of early recurrence in the control group is 48%. This change corresponds to an odds ratio of 0.4 (treatment vs control), which is nearly equivalent to the odds ratio observed in similar trials using colchicine (colchicine OR 0.38) at the significance level of 0.046 using a 1-sided Farrington and Manning likelihood score test, and a 48% rate of early recurrence in the placebo (control) group. The estimated 48% rate of early recurrence was extrapolated from our pilot data (unpublished work), which found a 45% rate of recurrence but did not use continuous monitoring as will be performed in our trial.

#### Recruitment {15}

Participants will be identified through the electronic medical record after being scheduled for an ablation. The study personnel will then discuss the study with the potential participant by phone, after approval from the clinical team. Vanderbilt University Medical Center is a large AF ablation referral center and typically performs 5–10 AF ablations per week.

## Assignment of interventions: allocation

### Sequence generation {16a}

A stratified permuted block scheme with a block size of 8 will be used for this trial. Participants will be stratified on the basis of three factors: (1) planned ablation technique (radiofrequency vs cryoballoon), (2) paroxysmal vs persistent AF prior to ablation, and (3) de novo vs repeat ablation for AF. The study statistician has designed and implemented the randomization table within a web-based platform that can be accessed by the study personnel. The randomization table will be unknown to all study personnel with the exception of the study statistician. The study statistician will remain blinded to the treatment, designated as “1” or “2.” The treatment assignments generated by the web-based platform will be sent to the personnel with the Vanderbilt Investigational Drug Service (IDS), who will allocate and ship the study drug to the participants directly.

### Concealment mechanism {16b}

The study drug will be started 3 days prior to ablation. At least 24 h prior to randomization, the study nurse will input the participant information necessary to stratify the randomization into the web-based platform and notify the IDS to randomize the participant and prepare the study drug. IDS will directly ship the intervention or matching placebo to the participant. MTI Biotech, Inc. (MTI) will provide 2-HOBA to the IDS as a 250-mg capsule. MTI will also provide matching placebo capsules. All participants will take 3 capsules every 8 h with identical pill bottle directions.

### Implementation {16c}

The study team will enroll participants by telephone call and electronic consent after identification as meeting the enrollment criteria. The study statistician designed and implemented the web-based randomization tool with inputs for the stratification factors as inputted by trial personnel. The study nurse will be responsible for inputting the stratification factors into the web-based tool, and the pre-identified permuted block randomization scheme as implemented in the web-based tool will assign treatment. This treatment assignment will be sent to IDS for the mailing of the capsules, either intervention or placebo.

## Assignment of interventions: blinding

### Who will be blinded? {17a}

All study personnel with the exception of the study nurse will be blinded to the treatment assignments and will not have access to the randomization tool. Blinding will be facilitated using tools within the REDCap data to restrict user access to specific data collection forms (e.g., treatment assignment and randomization tool). The study statistician designed and implemented the web-based randomization tool but remains blinded to the treatment assignments as listed assignments are listed only as “1” or “2.” Only the study nurse and the IDS will retain the association of the randomized assignment to the treatment assignment. These treatment assignments will be transmitted to the IDS which prepares and ships the placebo or intervention directly to the participants. All outcomes will be adjudicated by blinded study personnel who do not have knowledge of either the randomized designation (1 or 2) or the actual assignment (2-HOBA vs placebo). Participants will be blinded, with identical instructions listed for intervention and placebo. Care providers not associated with this study will similarly remain blinded as knowledge of the treatment assignment will be maintained only by the IDS and the study nurse.

### Procedure for unblinding if needed {17b}

The treatment assignment will be maintained by the IDS. If deemed clinically necessary, the unblinding may occur in case of a medical emergency or medical necessity should an adverse or serious event occur that is thought to be associated with the study drug. If a situation is so deemed, the IDS may be reached directly for treatment assignment. No other unblinding will be permissible, and participants will not be permitted to know the allocated intervention while the trial is ongoing. Following trial conclusion and data analysis, participants who wish to know may have their treatment assignments revealed by study personnel.

## Data collection and management

### Plans for assessment and collection of outcomes {18a}

Baseline participant characteristics will be collected directly from participant interview and the medical record. Given that these baseline participant characteristics are necessary for routine clinical care, missing data in baseline characteristics is anticipated to be minimal. For the primary outcome, participants will be instructed on the use of the wearable device twice while in the hospital for their ablation procedure. Part of this participant training involves the demonstration of the ability to take an ECG without further direction from the study personnel. All ECGs are automatically downloaded to the participant’s smartphone to be collected at the 1-month follow-up visit. ECGs will be reviewed by a board-eligible/board-certified cardiologist per standard clinical criteria. All tracings which meet the primary endpoint will be saved directly to a web-based platform, for review and confirmation at a later date by a second board eligible/board-certified cardiologist.

For the secondary clinical outcome, watch data will be correlated with a participant worksheet. Times in which the participant has self-recorded episodes of AF will be correlated with the wearable recordings. Wearable recordings will also be correlated with the total wear time of the wearable. For the biochemical assessment, blood samples will be obtained immediately prior to ablation and the morning of post-procedure day #1. They will immediately be processed for IsoLG adduct measurement. Single peripheral blood mononuclear cells (PBMCs) from each participant will be isolated initially by Ficoll density gradient centrifugation, as previously described [16]. IsoLG adducts in dendritic cells will be determined by flow cytometry using a single-chain antibody D11, which is labeled with a fluorochrome using the APEXTM AlexaFluor 488 Antibody Labelling Kit (Invitrogen). The cells labeled with surface antibodies will then be fixed and permeabilized for intracellular detection of IsoLG using a cell permeabilization kit (Invitrogen), as previously done [17].

### Plans to promote participant retention and complete follow-up {18b}

Per standard of care at our institution, all patients undergoing an AF ablation will have a 1-month clinical follow-up visit. This study involves the collection of 28 days of data following the ablation, correlating with the 1-month follow-up visit. Loss to follow-up within this 1-month period is expected to be rare due to high compliance with patients for their required post-procedure clinical follow-up.

Participants will be reminded to take a daily ECG both by the instruction sheet and reminders by the study nurse at follow-up encounters. The wearable also has a feature to alarm if a sustained elevated heart rate or irregularity in the pulse, possibly indicative of AF, is detected. Given that the participants will have just undergone an elective procedure for the purpose of reduction of AF, participant engagement is expected to be high.

### Data management {19}

All records will be retained on password-protected computers accessible only to members of the study team. Computers containing these records are only connected to the networks if they include appropriate firewalls and security measures. All study data will be maintained in the secure, web-based data entry tool, Research Electronic Database Capture (REDCap), which allows for secure data entry while on the secure hospital network [18]. Data values will be checked at the time of entry to ensure that data input is within range of reasonable values where applicable.

### Confidentiality {27}

The conduct of genetic studies raises specific issues with respect to the protection of human subjects. All records will be retained on password-protected computers accessible only to members of the study team. Computers containing these records are only connected to the networks if they include appropriate firewalls and security measures. Deidentified records and DNA samples may be shared with other investigators who have IRB-approved protocols and who agree to comply with the protections provided at this institution. These research materials will be transferred only by secure methods. The identity of any individuals and their families will not be revealed in any publication without their written informed consent.

### Plans for collection, laboratory evaluation, and storage of biological specimens for genetic or molecular analysis in this trial/future use {33}

The expected duration of this study is estimated to be 4 years. The study results will be retained for at least 6 years after the study is completed. Blood samples and genetic information will be kept for an undetermined period of time for future gene research.

## Statistical methods

### Statistical methods for primary and secondary outcomes {20a}

The primary clinical endpoint will test whether 2-HOBA reduces the rate of early recurrence of AF, atrial tachycardia, or atrial flutter within 28 days following AF ablation, collectively referred to as “AF recurrence.” The secondary biochemical endpoint will test whether 2-HOBA can reduce circulating IsoLG adducts on dendritic cells expressed as a percentage (IsoLG-adducted dendritic cells/total dendritic cells × 100). The exploratory secondary clinical endpoint will test the difference of AF burden in participants receiving 2-HOBA or placebo.

Participant demographics and other baseline characteristics will be listed by participant and/or summarized descriptively by treatment group and by stratification factors. Categorical data will be presented as frequencies and percentages. Binary and categorical data will be analyzed with Fisher’s exact test and chi-squared test. For continuous data, summary statistics will be presented. For single time point data, between-group differences will be assessed with the t-test (2 groups) or analysis of variance (3 or more groups) based on a continuous variable. Non-parametric counterparts, Wilcoxon rank sum test and Kruskal-Wallis test, will be used when assumptions for parametric methods are not met. Correlated data (e.g., repeated measurements) will be analyzed with generalized estimating equation (GEE) or linear mixed models (LMM). Multiple comparison issues will be corrected using Bonferroni’s approach. All statistical analyses will be performed using R 3.4.2 or a newer version.

The primary study hypothesis will test whether 2-HOBA reduces the rate of early AF recurrence of AF, atrial tachycardia, or atrial flutter following AF ablation within 28 days of follow-up. For simplicity, we will refer to this as “AF recurrence.” For the primary analysis, the full analysis set will be included in the primary analysis of AF recurrence, the primary efficacy endpoint. Binary recurrence of AF will be compared between treatment and control using a chi-square test. The primary analysis will be performed using a multivariable logistic regression model, where the dependent/outcome variable is the early AF recurrence (yes/no) by the end of the 28-day follow-up and the primary independent/predictor variable is the treatment arm (2-HOBA or placebo). Adjustment will be made for age, gender, AF type (paroxysmal vs persistent), method for PVI (radiofrequency vs cryoablation), additional ablation lesions (yes/no), BMI (continuous), and hours of monitoring (0–672 h). To avoid over-fitting our multivariable logistic regression model, a 10:1 ratio for degrees of freedom per the less frequent outcome event will be used in our modeling. Non-linear forms of the continuous predictors (age, BMI, and hours of monitoring) will be considered when building the model with restricted cubic splines. Models will be evaluated for goodness-of-fit and internally validated for the calibration and discrimination performance using the bootstrap method.

The exploratory, secondary analysis will analyze a surrogate of AF burden as the endpoint and be designed to account for the impact of cardioversion on AF burden assessment. AF burden is the time in AF, atrial tachycardia, or atrial flutter divided by the total time monitored. The secondary analysis will use the data provided by the continuous HR monitor provided by the smartwatch. The analysis will be performed using a linear mixed model (LMM) in which the AF burden is assessed weekly during the 4-week (28-day) follow-up period. The same covariates mentioned in the primary analysis will be considered. Additionally, AF burden will also be analyzed on an ordinal scale using a generalized estimating equation (GEE), ranked based on severity according to increasing AF burden and whether or not a cardioversion was performed. For any missing data in the covariates, we will use a multiple imputation method that incorporates predictive mean matching and flexible additive imputation models as implemented in the aregImpute function in the *Hmisc* R package. The impact of missing data on the validity of results will be examined with a pattern-mixture approach using the R package *SensMice*.

With regard to the biochemical investigation, the endpoint will be the IsoLG adduct content of dendritic cells expressed as a percentage (IsoLG-adducted dendritic cells/total dendritic cells × 100). We will test the hypothesis that 2-HOBA reduces the change in IsoLG adduct levels that occurs with AF ablation (as measured on post-procedure day #1 minus pre-ablation blood samples). Our previous pilot data (unpublished work) shows that the increase in dendritic cell IsoLG adduct level is 23% following ablation in our placebo (control) group, and we expect the range of possible observed reduction to be 20–40%. With *N* = 81 participants in each arm, the study provides at least 80% power to detect a minimal 22% reduction in lsoLG adduct level at a two-sided significance level of 5%. Our primary analysis will use multivariable linear regression to test whether our primary determinant (2-HOBA vs placebo) significantly reduces the change in IsoLG adduct levels (continuous), adjusted for baseline pre-ablation IsoLG adduct level and other potential confounding factors: age, sex, method for PVI (radiofrequency vs cryo), additional ablation lesions (yes/no), and BMI (continuous). The IsoLG content of dendritic cells adjusted for baseline level will be presented for both treatment arms, and together with corresponding 95% confidence intervals (CIs) and *p*-values will be graphically interpreted using boxplots, partial effect plots, residual plots, etc. A secondary endpoint will be the IsoLG adduct level in dendritic cells. Due to the prohibitively labor-intensive process required to measure IsoLG content in dendritic cells, only the pre-ablation and post-procedure day #1 samples will be analyzed for the analysis. However, blood samples from an additional time point (post-procedure day #28) will be available for pharmacokinetic analysis and secondary analysis of other serological markers. These will include malondialdehyde and other biomarkers of oxidative stress.

### Interim analyses {21b}

An interim analysis will be performed for superiority after half of the participants (*n* = 40 per group) have completed their 28-day assessment of the primary endpoint at the significance level of 0.006. The final analysis will be performed after all participants have completed their 28-day assessment of the primary point at a two-sided significance level of 0.041. Efficacy will be declared early at the interim analysis if the efficacy boundary is crossed (*p* < 0.007). All on-study participants will be followed for efficacy and safety until the planned end-of-follow-up. If the interim analysis indicates that the treatment is not better than the control by 13% (non-binding futility boundary *p* > 0.4), we may close the recruitment into the study with regard to any recommendations of the Data and Safety Monitoring Board (DSMB). The O’Brien-Fleming boundaries were used for the alpha- and beta-spending functions to preserve the overall type I and type II error. The interim analysis of serious adverse events will assess the number of serious adverse events that were determined to be possibly related to the study treatment (defined as “likely” or “not assessable”). If the upper bound is lower than 50%, the trial will continue; otherwise, we will terminate that arm.

### Methods for additional analyses (e.g., subgroup analyses) {20b}

Additional secondary analyses will include the following: (1) multivariable modeling of participants who were excluded from the primary analysis due to the use of colchicine or steroids during the follow-up period (will examine clinical and biochemical endpoints), (2) multivariable modeling with adjustment for 2-HOBA levels for the clinical and biochemical endpoints, and (3) multivariable modeling with adjustment for the duration of smartwatch use.

An exploratory genetic outcome seeks to explore the significance of variation at *PITX2*, a gene involved with the oxidative stress response [12]. DNA will be extracted, and genotyping will be performed on a genome-wide array. Imputation will be performed to a contemporary imputation panel that includes SNPs located at the AF susceptibility locus on chromosome 4q25. If the analysis was performed today, we would investigate rs2200733, rs17570669, and rs3853445. A 4q25 genetic risk score (GRS) will also be calculated to provide a weighted score of independent SNPs at the 4q25 AF risk locus. We hypothesize that a statistically significant interaction will exist between the 4q25 GRS and the treatment group (2-HOBA or placebo) for an association with our clinical and biochemical endpoints. In this exploratory genomics aim, we will therefore test for an interaction between the 4q25 GRS and the treatment group for an association with the primary clinical endpoint (early recurrence of AF). Adjustment will be made for the covariates used in the clinical endpoint. We will then add the 4q25 GRS to the multivariable linear regression model used in the biochemical endpoint and test whether there is a statistical interaction between the GRS and the treatment group for an association with the primary biochemical endpoint (change in dendritic cell IsoLG adduct level with ablation) by comparing models with and without the interaction term using likelihood ratio test. The added predictive value of 4q25 GRS and the interaction of GRS and treatment will be evaluated by comparing nested models with and without the predictor with regard to *c* statistics and the integrated discrimination improvement. Models involving 4q25 GRS will be internally validated using the aforementioned bootstrap method for the evaluation and correction of potential optimism in the performance of the model by balancing the bias and variance in the prediction error.

### Methods in analysis to handle protocol non-adherence and any statistical methods to handle missing data {20c}

The primary analysis for this study will be an intention-to-treat analysis. Crossover is anticipated to be low, as the intervention is not approved for use in AF and is not available at treatment doses over the counter. Similarly, the loss to follow-up and/or study withdrawal is similarly expected to be low due to the side effect profile of the study drug, the short duration of the study, and the regular follow-up at the 1-month interval for clinical care. Any participant withdrawn prior to completion of the 28-day visit/ascertainment of the primary outcome will be excluded from the primary analysis. A sensitivity analysis will be performed at the trial conclusion to evaluate the extent of withdrawal bias via an intention-to-treat analysis assuming all participants who withdrew recurred. For completeness, a second sensitivity analysis will be conducted as a “worst-case” analysis, assuming that all withdrawn participants randomized to 2-HOBA recurred and all those randomized to placebo did not. For any missing data other than the primary outcome, we will use a multiple imputation method that incorporates predictive mean matching and flexible additive imputation models as implemented in the aregImpute function in the *Hmisc* R package. Multiple imputation will not be employed if the primary outcome is missing; instead, the sensitivity analyses detailed above will be utilized. The impact of missing data on the validity of results will be examined with a pattern-mixture approach using the R package *SensMice*.

### Plans to give access to the full protocol, participant level-data, and statistical code {31c}

The protocol for this study is publicly available on ClinicalTrials.gov and submitted for publication here. Additional details will be provided upon request as necessary. Full statistical code for analysis will be published with the final publication of the trial results when completed. De-identified participant-level data may be available upon reasonable request, after request review by the study team and the local institution.

## Oversight and monitoring

### Composition of the coordinating center and trial steering committee {5d}

As a single-site, investigator-initiated clinical trial, we will not have a coordinating center or trial steering committee.

### Composition of the data monitoring committee, its role, and reporting structure {21a}

Oversight of the data and safety monitoring plan will be provided by a DSMB. The DSMB will meet at least twice a year and review data on AEs, adverse drug reactions, data quality, and study recruitment. DSMB reports will be sent to the IRB at least yearly. All members of the DSMB are independent of the study and independent from the investigational drug supplier. The DSMB will review all SAEs. Any SAE will be reported to the DSMB, IRB, and FDA as soon as possible, but not more than 3 days from the investigators’ awareness of the event. Any untoward medical event will be classified as an AE, regardless of its causal relationship with the study.

### Adverse event reporting and harms {22}

Any AEs will be reported to the PI within 72 h of notification of the event. The PI will notify the DSMB of any major AEs. Any unanticipated problems involving risk to the participants or others will be discussed with the PI and DSMB. Non-serious AEs and incidences of non-compliance with the protocol will be reported to the IRB at the time of the annual review. SAEs will be reported according to the following procedure: the occurrence of SAEs will be reported to the PI within 24 h after notification of their occurrence. The investigator will report SAEs to the DSMB and the Vanderbilt University Medical Center IRB within 7 days of the investigator’s notification of the event. In an unanticipated event of prolonged side effects, requiring prolongation of hospital stay, participants will be retained in the hospital until the side effects have resolved. For minor side effects, wherein participant care is deemed unnecessary, follow-up will be maintained via phone or as outpatient if necessary. Participant and their families will be given the PI’s contact number for reporting any other effects of medication following discharge. Any newly discovered information which may affect the participant or their caregiver’s decision to continue to participate in the study will be passed on to them as soon as possible. This may also result in a change to the consent form and review by the IRB.

### Frequency and plans for auditing trial conduct {23}

The DSMB will meet every 6 months or every 30 participants (whichever is sooner) to review the trial conduct and ensure protocol compliance. The DSMB is composed entirely of independent members who are not associated with the supplier of the drug. The DSMB members similarly are not members of the investigator team.

### Plans for communicating important protocol amendments to relevant parties (e.g., trial participants, ethical committees) {25}

Any protocol modifications must be approved by the DSMB and IRB prior to the implementation in this trial. Protocol modifications which affect participants directly after enrolling in the trial will be communicated directly to participants via the study nurse. This is an investigator-initiated and sponsored study; all protocol changes will be implemented by the investigator team directly.

### Dissemination plans {31a}

The current trial is registered as NCT04433091. Results from the trial will be disclosed via publication at the end of the trial following data analysis, as well as applicable national meetings.

## Discussion

Although oxidative stress and inflammation are major mechanisms contributing to AF pathogenesis, therapeutic targeting of these mechanisms to date is limited. Prior attempts targeting inflammation and oxidative stress for the treatment of AF using typical antioxidants such as fish oil have shown low efficacy [19]. While anti-inflammatories such as steroids and colchicine have been shown to reduce AF recurrence following catheter ablation, unfavorable side effect profiles have precluded their long-term use. With the discovery of IsoLGs and their role in cellular damage from oxidative stress, the availability of pharmacological agents such as 2-HOBA to bind IsoLGs has generated considerable interest as a novel therapy for AF. This trial will thereby test the clinical efficacy of a novel means to reduce AF recurrence in a high inflammation state.

If the proposed trial shows benefit for AF reduction, further investigations could assess the utility of 2-HOBA for the prevention of AF in additional populations, either in similarly high-inflammatory states (e.g., post-cardiac surgery) or more broadly for prevention of AF in the general population. The mechanistic rationale behind the results of this trial may have implications for therapy for diseases of oxidative stress more broadly. Delineating observed effects between the clinical outcome of reduced AF burden and the biochemical outcome of IsoLG reduction represents a key point of the proposed study.

This trial proposes the use of a wearable smartwatch for the detection of AF. Rapidly advancing smartwatches and other wearable technologies hold the potential to significantly impact the practice of medicine [20]. The Apple Watch series 4 has been validated to detect irregular pulses with its plethysmography sensor, as shown in a recent study monitoring 419,297 participants [13]. This trial proposes to use the on-demand ECG functionality of the Apple Watch to provide a point-of-care rhythm strip for adjudication of the primary outcome. Additionally, we are exploring the use of the Apple Watch as a means of quantifying AF burden in an exploratory secondary analysis. Although the burden of AF is thought to be a more clinically meaningful endpoint [15] for effective therapy for AF, measurement of AF burden has proven difficult. While the burden decreases after PVI [21], the measurement is highly skewed [22], and not all patients have continuous monitoring devices. The Apple Watch may afford the opportunity to have more readily available, semi-continuous monitoring for patients with AF, if the burden can be quantified in a clinically meaningful way as this trial proposes to do.

As a phase II trial, the direct clinical impact of this work remains limited. If positive, the mechanistic emphasis of this trial highlights the importance of a potential new class of therapeutic agents to attenuate inflammation and oxidative stress contributing to AF. However, based solely upon the result of this trial, 2-HOBA is unlikely to be either approved or utilized as a therapy for AF without further phase III trials. While the reduction of AF recurrence after ablation via a novel mechanism may highlight the promise of IsoLG scavengers for therapeutics, AF recurrence within the blanking period is of modest clinical impact. Reduction of recurrence of AF in the blanking period may reduce morbidity and cost related to hospitalizations and cardioversions associated with these recurrences; however, this trial is not powered for these outcomes. Additionally, the current 2-HOBA dosing regimen of three pills three times per day may affect compliance. As a mechanistic study, this limitation would need to be addressed by compounds with improved pharmacokinetics for future phase III trials.

The public health burden of AF demands new approaches for the treatment. The proposed trial will investigate the use of 2-HOBA to attenuate oxidative stress in AF and provide both biochemical and clinical insights that will guide further development of this novel class of IsoLG scavengers.

## Trial status

The current protocol is Serial 001 as submitted in an IND to the FDA on January 2, 2020. Recruitment began on June 1, 2020, and 22 participants are currently enrolled. Target enrollment is 162 participants with an expected completion date of Spring 2022.

## Data Availability

The datasets used and/or analyzed during the current study are available from the corresponding author on reasonable request.

## Abbreviations

2-HOBA: 2-Hydroxybenzylamine
AAD: Anti-arrhythmic drug
AE: Adverse event
AF: Atrial fibrillation
GEE: Generalized estimating equation
GRS: Genetic risk score
IDS: Investigational Drug Service
IRB: Institutional Review Board
IsoLG: Isolevuglandin
LMM: Linear mixed model
MTI: MTI Biotech, Inc.
PVI: Pulmonary vein isolation
REDCap: Research Electronic Database Capture
ROS: Reactive oxygen species
SAE: Serious adverse event
